# The law and problematic marketing by private umbilical cord blood banks

**DOI:** 10.1186/s12910-020-00494-2

**Published:** 2020-07-01

**Authors:** Blake Murdoch, Alessandro R Marcon, Timothy Caulfield

**Affiliations:** Health Law Institute, Faculty of Law, University of Alberta, Edmonton, Alberta T6G 2H5 Canada

**Keywords:** Umbilical cord blood, Biobanking, Misleading marketing, Health law

## Abstract

**Background:**

Private umbilical cord blood banking is a for-profit industry in which parents pay to store blood for potential future use. Governments have noted the tendency for private banks to oversell the potential for cord blood use, especially in relation to speculative cell therapies not yet supported by clinical evidence. We assessed the regulatory landscape governing private cord bank marketing in Canada.

**Main body:**

Because the problematic marketing of private cord blood banking for future use often relates to speculative future cell therapies that do not exist and are not being advertised for current clinical use, most private blood bank marketing seems to fall outside Health Canada’s regulatory scope. However, this problematic marketing is regulated by the Competition Bureau pursuant to the *Competition Act.* While representations relating to future hypothetical treatments may not always be subject to the legal requirement for claim substantiation, the law also prohibits individuals and companies from knowingly or recklessly making representations that are “false or misleading in a material respect.” A representation is materially false or misleading when it could “influence a consumer’s behavior or purchasing decisions,” and consumers are likely to be considered to be “credulous and inexperienced” for the purposes of assessing an advertisement’s general impression. Because all of the potential benefit of the banking is derived from the potential future use of the biological material for health interventions directed toward the customers and their relatives, and because we know the best available medical evidence indicates a very low probability of utility in this context, we can say with confidence that some private cord blood banking claims are materially misleading. Moreover, to the extent that medical professionals are involved in private bank interactions with customers or hold ownership stakes in private banks, they are subject to professional codes, standards of practice, and potentially fiduciary obligations that further prohibit misleading marketing.

**Conclusions:**

Private cord blood bank marketing that advertises hypothetical future treatments can be misleading and may influence consumer behaviour. This marketing may breach existing advertising law. Regulatory bodies should enforce the law in order to help prevent public health and personal financial harm.

## Background - the problem

Umbilical cord blood is a rich source of stem cells that can be used in treatments for blood and immune system disorders. It is also used for research into novel therapies not yet ready for clinical application [1, 2]. The use of the stem cells in these applications depends on individuals banking their cord blood in either public, private, or public-private hybrid banks. It is an individual’s choice to bank their cord blood and to do so in either a public or private bank. That decision has the potential to be influenced by numerous sources, including health care practitioners and family and friends [3]. But the media – including the popular press, social media, and communication efforts of both public and private entities – can also have an impact [4–7]. Research shows that there are issues around the public’s awareness and knowledge of cord blood uses, and that information sources are “varied, fragmented and inconsistent” [3].

Private banking is a for-profit industry through which parents pay to store umbilical cord blood for potential future use. There are currently seven private banking companies marketing in Canada, typically charging an initial fee of approximately $1000.00 CAD, followed by a yearly charge of approximately $125.00 [8]. Public banking is a form of donation where the tissues are generally used to help other patients or for research, is free of cost, and is overseen by government agencies. In Canada, the first private banks began operation in 1996 [9]. With the exception of Quebec, which began public banking in 2004 [10], there was little public banking in Canada until the creation of the national program in 2013 [11]. In this respect, Canada was significantly behind other countries, such as, for example, the United Kingdom, which had an operational public bank program in 1996 [12], and the United States which established its National Cord Blood Program in 1992 [13]. In Canada and abroad, policy statements and government discourse often recommend public cord blood banking over private banking, except in cases of an established prior condition in the family that could benefit from cord blood use [1, 14–18]. This is because the potential for autologous or familial allogeneic use offered by private banking is extremely low (a point confirmed by usage data) [1], private banking is costly, and it can elude regulatory oversight, in turn impacting overall blood quality [1]. In contrast, public banks provide public health benefits and regulatory oversight helps to ensure the effectiveness of processes while minimizing risk [1, 14, 15, 19].

Provincial and federal governments stress the tendency for private banks to oversell the potential for cord blood use, especially in relation to speculative stem cell therapies not yet supported by clinical evidence [14–16, 19]. Academic research has described public banks as operating on a “regime of truth” but characterizes private banks as operating on a “regime of promise.” [20, 21] Private banks can wield the power of hope to their marketing advantage, exacerbating problematic “promissory” [22] and “hyping” [23–26] aspects of health science. For example, as of June 8, 2020, one public bank stated on its website contained a news link to a story indicating that a “boy with brain injury” showed “‘remarkable’ improvement after experimental stem cell procedure”, also stating that its services offer “the opportunity to collect and save your baby’s cord blood stem cells for potential medical uses to treat life-threatening diseases (createcordbank.com). Another bank claims that its services are a “once in a lifetime opportunity to protect your child’s future health” (futurehealthbiobank.com/ca-en). The factual divisions between public and private banking options are expressed on popular websites likely to be accessed by future parents (i.e. https://www.babycenter.ca/ [27] or www.webmd.com [28]), and private banking marketing initiatives have received ample coverage in popular Canadian press [29–32].

Recent research shows that, in the North American popular press, the topics of public and private banking feature significantly when cord blood is discussed [33]. Overall, private cord blood banking is portrayed more in relation to concerns/risks than benefits, and strong narrative messaging often accompanies this portrayal [33, 34]. As discussed in the academic literature, it is common for private banking to be marketed as a type of “biological insurance” used by parents wishing to do the very best for their children and families [20, 29, 33, 35]. This is noted when private companies label themselves as “family” banks [8, 20, 29, 35]. On the other hand, donating blood to public banks can often be an unacknowledged process, and some have argued that it requires significant improvement [21, 36–39]. Ultimately, an individual’s decision on what to do with cord blood can largely hinge on obtaining accurate, up-to-date knowledge. Private banking marketing efforts, including information provided on their websites, can significantly influence decision making. Specifically, they can potentially take advantage of [future] parents’ strong desire to protect existing and/or future children.

Here, we assess the regulatory landscape governing private cord bank marketing in Canada, with a focus on determining the permissibility of advertising claims relating to future, as yet unproven therapies that would use banked cord blood.

## Main text

### Health Canada

Health Canada’s role in the regulation of private cord blood banking depends on the functions banks choose to perform. Private banks that store for allogeneic use (i.e., use in a different individual) are subject to routine inspections and the Safety of Human Cells, Tissues and Organs for Transplantation Regulations [16, 40]. However, direct-to-consumer marketing usually pertains to private cord blood banking for future autologous or familial allogeneic use, and these banks are regulated under the Food and Drugs Act [16, 41]. Through this and the Blood Regulations, cord blood “products cannot be manufactured, prepared, preserved, packaged or stored under unsanitary conditions.” [16, 42]

As was noted in a recent position paper released by Health Canada, all cell therapies are “drugs” as defined in the Food and Drugs Act, and are regulated under it and the associated regulations [43, 44]. This means that any cord blood therapies used by banks or their partners in the future would need to complete the clinical trial process and be approved by Health Canada. Institutions wishing to perform treatments in the future would be limited to approved therapies, and would not be able to perform unproven or unapproved therapies in the manner private clinics recently have in the context of stem cell therapies [45–47].

Health Canada has the ability to regulate marketing of drugs and medical devices pursuant to its jurisdiction [48]. The service of private blood banking is neither a drug nor a medical device [49]. Also, because the marketing of private cord blood banking for autologous use often relates to speculative future cell therapies that do not exist and are not being advertised for current clinical use, most private blood bank marketing seems to fall outside Health Canada’s regulatory scope. As such, we must turn to other areas of the law for instructive input.

### Truth in advertising

The marketing practices of private cord blood banks in Canada are regulated by the Competition Bureau pursuant to the *Competition Act* [50]. There are also industry standards for advertising, though they are not legally binding. Ad Standards has the *Canadian Code of Advertising Standards*, a code of conduct which is based in substance on underlying advertising law [51, 52]. Ad Standards also has a voluntary complaint system through which a council reviews concerns and renders decisions, which can then be appealed if desired [53]. Though the institution lacks significant enforcement capability, it can report the improper marketing behaviours of uncooperative companies directly to the Competition Bureau.

Under the *Competition Act*, individuals and companies are prohibited from knowingly or recklessly making representations that are “false or misleading in a material respect.” [50] All performance claims must be supported by an “adequate and proper test” showing that the advertised product or intervention has the claimed effect. This means that some evidence must be available to support the marketing claims. However, under existing truth-in-advertising precedent, that test does not have to follow a rigorous scientific process [54]. The test only requires establishing that a product has an effect beyond chance [54].

Similar to Health Canada’s noted inability to regulate these claims, the applicability of the truth-in-advertising law relating to claim substantiation to the unproven future therapies predicted in private cord bank advertising is questionable. This is because the actual product or service offered is the banking of blood, not the predicted or hypothesized future treatments that may be mentioned in marketing. It is possible the suggestion that unproven hypothetical future treatments could make blood banking a great investment for one’s future health may not always trigger regulatory oversight. This could depend on the extent to which future therapies are hypothesized rather than claimed to be inevitable or guaranteed. In this sense, private cord banking marketing is distinct from directly advertising the benefits of unproven therapies, as the latter clearly requires substantiation of a performance claim under the requirements set out by the Competition Bureau and enabling law [55].

Yet, while the representations of private cord blood banks relating to future hypothetical treatments may not always be subject to the legal requirement for substantiation, the main provision of the *Competition Act* prohibiting false advertising still appears to be directly applicable. As noted, section 74.01 states promotional content is reviewable if it is “false or misleading in a material respect.” [50] The standard of materiality is met when the “literal meaning or general impression conveyed could influence the ordinary consumer to buy or use the advertised product or service” [56] – that is to say, when it could “influence a consumer’s behavior or purchasing decisions.” [51] (Notably, this is very similar to the United States’ standard for deceptive practices, which is met if a practice is likely to mislead consumers and “affect consumers’ behavior or decisions about the product or service” [57]; it is also similar to the United Kingdom’s standard for misleading advertising, which is met when marketing, “by reason of its deceptive nature, is likely to effect [traders’] economic behavior” [58].) The Supreme Court of Canada has addressed the legal meaning of a general impression in the context of Quebec’s *Consumer Protection Act*, a provincial statute that is largely based on the *Competition Act*. In *Richard v. Time, Inc,* the Supreme Court held that assessing a general impression requires “describing the general impression that the representation is likely to convey to a "credulous" and inexperienced consumer; and [2] determining whether that general impression is true to reality” [59].

While this precedent has not yet been reaffirmed in federal *Competition Act* proceedings, it is highly instructive of the manner in which general impressions will be assessed federally. The case suggests that consumers are, for the purpose of assessing truth-in-advertising claims, to be considered to be “credulous and inexperienced.” This means that the standard for a “general impression” to be considered misleading is low. Statements about potential future health benefits from banking blood that do not currently exist, if they form part of the general impression of a private bank’s representation, could easily mislead a "credulous" and inexperienced consumer – especially in the emotional context of pregnancy or birth.

Further, the private banking of cord blood is generally not, solely as a service in itself, of any use or benefit to a customer/patient. Generally, all of the potential benefit of the banking is derived from the potential future use of the biological material for health interventions directed toward the customer/patient or his or her relatives. Therefore, the crux of the service, that is to say what the private bank is “really selling,” is the potential for future health benefit. Indeed, a slightly improved storage technology – that is to say a measurable improvement to the de facto service offered – is arguably much less likely to sway consumer behavior than, for example, the promise of a future stem cell therapy that could cure a loved one. Since the potential for future health benefit is the key reason why private banking exists, it is very likely that a court would find claims about future health breakthroughs and technologies to be material to consumer decision making. And when we consider that the best available medical evidence indicates a “low probability of using one’s own cord blood for autologous transplantation, which studies have estimated are between 1 in 20,000 and 1 in 250,000,“ [16] we can say with confidence that some private cord blood banking claims are materially misleading.

In sum, truth in advertising law remains the most valuable enforcement tool for claims about hypothetical future treatments for which scientific support is preliminary or non-existent. Nonetheless, the Competition Bureau will need to engage in enforcement to effectively minimize this sort of advertising. Enforcement should be feasible given that only seven private companies operate in Canada, though public complaints would certainly aid in spurring the Bureau into action.

### Other relevant law and policy

In addition to truth in advertising law, the common law relating to negligence, misrepresentation, and fiduciary obligation can be helpful in contexts where health professionals are presenting banking options to patients. Common law requires that these professionals practice to the standard of “a prudent and diligent doctor in the same circumstances,“ [60] a standard that is generally incompatible with interventions that are not evidence-based [46, 61]. Physicians also have a legal duty of care to patients who are making medical decisions, and pursuant to this duty they must disclose all information a reasonable person in the patient’s position would want to know [62, 63]. While the service of preserving cord blood is itself evidence-based, advertising for yet unknown future therapies is not. Hence, disclosure requirements likely mandate that physicians inform patients about the problems with these representations, and the facts and probabilities relating to the low chance of utility from private banking. In Canada, based on the statements of provincial governments such as those in Alberta and British Columbia, “doctors do not recommend that you bank cord blood on the slight chance that your baby will need stem cells someday,” and patients should “consider donating the cord blood to a public bank instead.” [14, 15]

For most health-related advertising, health professional standards of practice, codes of ethics and disciplinary tribunal precedents are applicable and set a high standard of conduct [46, 64, 65]. This is because marketing by health professionals often relates to a medical intervention or a health product used directly by a patient on their body, which triggers both the noted legal obligations but also these standards. However, the applicability of these policies could be questionable in the context of private cord blood bank marketing. To our knowledge, there is no requirement that patients consult with a bank’s health professionals prior to entering into an agreement to store cord blood with a bank, and while physicians and other medical professionals may hold positions at various levels in a bank’s business structure, client contact is likely to be minor and not to constitute a physician/patient relationship that would trigger additional professional standards and obligations. Still, if a physician is involved, he or she would be bound by relevant standards and ethical requirements regarding advertising, and if a private bank were under a physician’s ownership it is also likely these standards would apply.

## Conclusions

When making decisions about biobanking, consumers must be presented with accurate information about the likelihood of private banking resulting in benefit. Disclosures should also include a consideration of the opportunity cost of storing privately instead of donating to a public bank and possibly contributing to a life-saving therapy for someone else. Both public health and personal financial harm can occur when misleading advertisements are used to convince patients to use a private instead of a public cord blood bank.

Here we have argued that private cord blood banking advertising that includes claims relating to speculative future health benefits from unknown or unproven therapies potentially breaches advertising law. Though we have used Canada as an example, and there may be legal differences in other jurisdictions that manifest as slightly different limits on the extent of permissible prognostication, the general proposition holds that such marketing is materially misleading in a way that is likely to impact consumer decision making – and especially the decision making of a "credulous" and inexperienced consumer [59]. Regulatory bodies such as the Competition Bureau, industry associations such as Ad Standards and non-profit advertising watchdogs such as Truth in Advertising should all act to encourage enforcement of existing law, to help prevent new parents from being persuaded to privately store tissue that is likely of no personal utility and may result in significant financial and public health cost.

## Data Availability

Not applicable.
